# A Case of Ulcerative Colitis During Pregnancy and Childbirth at an Older Age While on Biologic Therapy

**DOI:** 10.7759/cureus.66689

**Published:** 2024-08-12

**Authors:** Tomotaka Tanaka, Daiki Hirano, Syohei Ishimaru, Keiko Arataki

**Affiliations:** 1 Department of Gastroenterology, Tsuchiya General Hospital, Hiroshima, JPN

**Keywords:** infliximab, older births, older pregnancies, ustekinumab, ulcerative colitis (uc)

## Abstract

There is considerable uncertainty regarding the safety and efficacy of biological therapies during pregnancy. We report a case of a 46-year-old female, diagnosed with ulcerative colitis over 30 years ago, who was successfully managed with infliximab and ustekinumab. She experienced no exacerbation of her condition during two pregnancies, demonstrating the safety of biologic therapy in maintaining disease remission during pregnancy. This case report highlights successful outcomes despite the uncertainties associated with biologic therapy during pregnancy and includes a brief review of the relevant literature.

## Introduction

Ulcerative colitis (UC), an inflammatory bowel disease (IBD), is a chronic, intractable, inflammatory bowel disease of unknown cause that repeatedly relapses and goes into remission. Because UC is prevalent in young people, it often overlaps with the age of pregnancy and childbirth in women. In recent years, the number of UC patients has increased, and we have seen more cases of women hoping to have children, successfully becoming pregnant, and giving birth. Recently, biologics have become available that dramatically control the condition by inhibiting interleukins and tumor necrosis factor α, which are involved in inflammation. This suggests an increase in the number of UC pregnancies and childbirths during biologic treatment.

Here, we report a case of a UC patient who became pregnant and gave birth at advanced ages, 39 and 46 years, while undergoing biologic treatment. We also present a literature review.

## Case presentation

Case

A patient developed ulcerative colitis in 1991. The disease type was pancolitis, and the clinical course was classified as relapsing-remission type. The severity at onset was mild to moderate, and the total amount of steroids administered from onset to the present was approximately 10,000 mg.

Clinical course

From 1991 to around 2002, remission was induced and maintained by oral administration of 5-aminosalicylic acid (5-ASA) alone. From around 2002, remission was induced by oral administration of steroids whenever symptoms recurred. However, relapses became more likely when steroids were reduced or discontinued (steroid dependence). Due to steroid-induced adverse events, including a full moon face, steroid administration was discontinued around 2004, and granulocytapheresis (GMA) was initiated. From 2009 to 2013, she started oral tacrolimus treatment, and GMA was used in combination with the treatment to induce and maintain remission.

In 2013, frequent visits to the hospital became difficult, so infliximab (IFX) was used as induction therapy, and after remission induction, IFX was administered every seven to eight weeks for remission maintenance. She got married in 2015. In 2016, at the age of 39, she became pregnant with her first child. She continued IFX during pregnancy and childbirth (normal vaginal delivery, female 2724 g) considering the possibility of UC relapse, but there was no UC relapse due to pregnancy or delivery, and no abnormalities in delivery. Since IFX became ineffective later, she switched to ustekinumab (UST) in 2020. UST was administered at approximately eight-week intervals to maintain remission.

**Figure 1:**
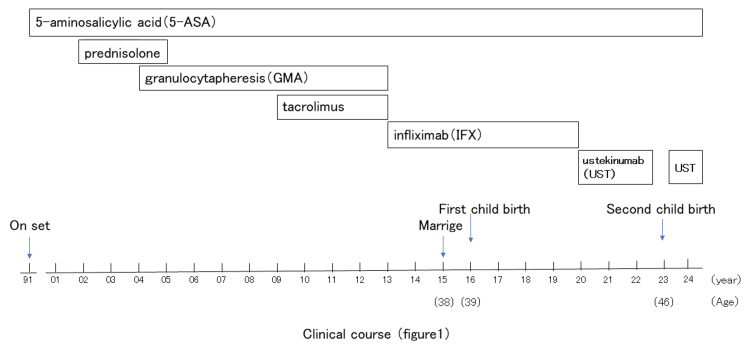
Clinical course

In 2023, at the age of 46, she became pregnant with her second child. UST was discontinued at 30 weeks of pregnancy. Due to placenta previa, a cesarean section was scheduled at 36 weeks, and the baby was delivered (normal, female, 2248 g). There was no recurrence of UC or abnormal delivery. UST was resumed seven weeks after delivery, and one year has passed since delivery without any recurrence of UC and both mother and child are doing well (Figure 1).

## Discussion

It is well-known that there are many cases where inflammatory bowel disease (IBD) remains in remission during pregnancy but recurs after childbirth. No definitive conclusions have been reached regarding the impact of pregnancy and childbirth on the condition of IBD (UC). Previous reports have suggested that overseas, the annual relapse rate of UC in non-pregnant women is 32%, while the relapse rate during pregnancy is 34%, with no difference in relapse rate [1]. However, in Japan, Kobayashi et al. reported that relapse occurred in 17 of 36 pregnancies during UC remission (47.2%), suggesting that pregnancy may be a factor in UC relapse [2]. Oriuchi et al. reported that pregnancy has little impact on UC in a study of 47 cases and 70 pregnancies complicated by UC [3], and Endo et al. reported that the relapse rate in pregnancies during UC remission was 65.2%, with the impact of pregnancy and childbirth on UC relapse during remission being somewhat smaller than the UC relapse rate at their facility (63.1%) [4]. Oriuchi et al. recommend pregnancy after at least one year of remission, stating that if pregnancy is achieved after maintaining remission for more than one year, the relapse rate during pregnancy and activity during relapse are low. Our previous report of 22 cases of UC also suggested that pregnancy during remission is preferable [5].

Regarding the impact of IBD (UC) on pregnancy and childbirth, many overseas reports state that the incidence of miscarriage, premature birth, low birth weight, and congenital abnormalities in newborns is higher than in normal pregnancy and childbirth [6-9]. Mahadevan et al. reported that conception abnormalities (miscarriage) were 23%, pregnancy abnormalities (premature birth, low birth weight, and stillbirth combined) were 25%, and congenital abnormalities in newborns were 7% [6]. However, in Japan, Oriuchi et al. reported that UC has little impact on pregnancy and childbirth except in severe cases [3], and Endo et al. reported that the rate of pregnancy and childbirth abnormalities in UC is 30.6% [4]. There was no consistent trend in the specific types of pregnancy and birth abnormalities. Miscarriage, premature birth, placental abnormalities, low birth weight babies, and congenital abnormalities in newborns were observed sporadically, but their frequency was roughly the same as or lower than the frequency of non-UC pregnancies (miscarriage 14%, premature birth 9.6%, stillbirth 0.2%, placental abnormalities 1%, low birth weight babies 10%, congenital abnormalities in newborns 6%). This suggests that UC has little impact on pregnancy. Many reports suggest that pregnancy and birth during remission are desirable to avoid pregnancy and birth abnormalities [4,5,10]. According to the Ministry of Health, Labour and Welfare Sciences Research Grant (Intractable Disease Policy Research Project) on Pregnancy Complicated with Inflammatory Bowel Disease, of the 104 study participants, 24 cases of Crohn's disease and 70 cases of ulcerative colitis were registered. Among them, 59 were live births, 6 were miscarriages, and there were no stillbirths. There was one case of congenital abnormalities, five cases of premature birth, and nine cases of low birth weight less than 2500g (1886g-2480g), although these were not significantly higher than the rate of complications in the general population [11].

The administration of IBD medications during pregnancy is typically undertaken when the benefits of managing IBD symptoms in the mother are considered to outweigh the potential risks of adverse effects, teratogenicity, and fetal toxicity. Regarding the safety of UC drugs, a study of 113 cases and 207 IBD-complicated pregnancies in Western literature reported no correlation between the use of 5-aminosalicylic acid (5-ASA), steroids, immunomodulators, antibiotics, and other drugs and the occurrence of pregnancy and birth abnormalities [10,12]. Endo et al. also reported no difference in the frequency of pregnancy and birth abnormalities between the use and non-use groups of 5-ASA and systemic steroid administration [4].

The most useful reference for the use of 5-ASA and biological agents during pregnancy is the "Treatment guidelines for pregnancy and birth for female patients with systemic lupus erythematosus (SLE), rheumatoid arthritis (RA), juvenile idiopathic arthritis (JIA), and inflammatory bowel disease (IBD)" [13]. Both 5-ASA and IFX are considered relatively safe (level of recommendation: B/level of agreement: [8]. However, since biological agents are known to be transferred through the placenta during the late stages of pregnancy (after 30 weeks) and to be transferred to the newborn's blood, some reports suggest that they should be avoided during the late stages of pregnancy [12,14,15]. This is because the effects of IFX and UST present in the newborn's blood on the development of the newborn's immune system have not yet been fully elucidated.

In addition, if IFX or UST is administered in the late stages of pregnancy, the question of whether or not to vaccinate the newborn after birth is also a major issue. It is unclear whether vaccination will adequately acquire immunity, and there is concern about the risk of infection in newborns from live vaccines. In a report of a Crohn's disease case in which IFX or UST was administered until the late stages of pregnancy, IFX was detected in a 6-month-old infant, but UST had maternal blood concentrations of 267.7 ng/ml and umbilical cord blood concentrations of 756.5 ng/ml but was not detected in a 6-month-old infant [15], suggesting that UST may have less of an effect on newborns than IFX. The "Treatment guidelines for female patients with systemic lupus erythematosus (SLE), rheumatoid arthritis (RA), juvenile idiopathic arthritis (JIA), and inflammatory bowel disease (IBD) during pregnancy and childbirth" also reports that, although anti-TNFα antibody preparations can be used throughout pregnancy if used up until the end of pregnancy, there is a risk of influence on the baby due to placental transfer. Therefore, it is better to refrain from administering live vaccines, such as BCG and rotavirus vaccines, before the baby reaches six months of age (level of recommendation: B/level of agreement [8,13].

In this case, we experienced a long-term UC case where the patient became pregnant and gave birth to her first child while receiving IFX maintenance therapy at age 39, and became pregnant and gave birth to her second child while receiving UST maintenance therapy at age 46.

In this case, colonoscopy was not performed before pregnancy, so it was unclear whether the UC had been cured by mucosal healing. Considering the clinical course, IFX was continued at 7 to 8 weeks because the patient had repeated relapses. Given the high risk of UC relapse at the age of 39, we consulted with the patient and continued the pregnancy and delivery while maintaining IFX. There was no UC relapse after delivery, but we instructed the patient to refrain from receiving the BCG vaccine, which is usually administered at five to seven months, and the rotavirus vaccine, which is usually administered at two to four months, until six months after delivery.

At the age of 46, the patient was even older than when she became pregnant and gave birth to her first child and was receiving UST (anti-IL-12/23p40 monoclonal antibody preparation) instead of IFX (anti-TNFα preparation). The safety of UST has not yet been established in the literature, so taking into consideration the effects on the mother and fetus, UST was discontinued at 30 weeks, and the patient gave birth. At present, there have been no confirmed cases of postpartum UC recurrence or effects on the fetus. However, the effects of UST on the newborn cannot be denied, so as a precaution, we instructed the patient to refrain from administering live vaccines for six months after delivery, as was done previously.

Both pregnancies and births were unexpected, so in the future, we will have to constantly consider this possibility when providing medical care. Regarding UC treatment during pregnancy, although it depends on the patient's condition, there seems to be no safety issue in continuing the administration of biological agents even at an advanced age to become pregnant and give birth during the remission period. However, there are few case studies and literature reports on the effects of biological agents on the fetus, so further investigation is needed.

## Conclusions

We report a case of an elderly pregnant woman with ulcerative colitis who was receiving biologic therapy. The biologic therapy maintained remission of ulcerative colitis, allowing her to become pregnant and give birth relatively safely. This treatment appears to be beneficial for women of childbearing age, not just those with advanced age.
